# Modified* Si-Ni-San* Decoction Ameliorates Central Fatigue by Improving Mitochondrial Biogenesis in the Rat Hippocampus

**DOI:** 10.1155/2018/9452127

**Published:** 2018-07-29

**Authors:** Chenxia Han, Feng Li, Yan Liu, Jie Ma, Xue Yu, Xiumei Wu, Weiyue Zhang, Danxi Li, Dou Chen, Ning Dai, Bingqi Lin, Fengzhi Wu, Meng Mao

**Affiliations:** ^1^Basic Medicine School, Beijing University of Chinese Medicine, Beijing, China; ^2^Department of Integrated Traditional Chinese and West Medicine, West China Hospital, Sichuan University, Chengdu, Sichuan, China; ^3^Insect Biological Medicine Research Institution, Dali University, Yunnan, China

## Abstract

The traditional Chinese medicine (TCM) decoction* Si-Ni-San* (SNS) has been utilised for millennia to improve physiological coordination of the functions of the liver and spleen, which are regarded as the main pathological organs of central fatigue in TCM. This study evaluates the effect of a modified SNS (MSNS) formula on central fatigue in rats and explores molecular changes associated with hippocampal mitochondrial biogenesis. Central fatigue was induced through a 21-day sleep deprivation protocol. We assessed MSNS's effects on behaviour, blood and liver biomarkers, and mitochondrial ultrastructure. We found that MSNS could reverse various signs of central fatigue such as its effects on hippocampal gene and protein expression levels of sirtuin 1 (SIRT1), peroxisome proliferator-activated receptor gamma coactivator-1*α* (PGC-1*α*), and nuclear respiratory factor 1 (NRF1). We also observed evidence of MSNS decreasing central fatigue, such as decreasing creatine kinase activity, decreasing levels of malondialdehyde and blood urea nitrogen, increasing lactate dehydrogenase and superoxide dismutase activities, increasing mitochondrial DNA copy number, and reversing mitochondrial ultrastructure changes. These findings suggest that MSNS can ameliorate central fatigue and that its molecular mechanism involves mitochondrial biogenesis enhancement mediated by hippocampal SIRT1, PGC-1*α*, and NRF1.

## 1. Introduction

Central fatigue (CF) is the failure to initiate and sustain attention and physical activities requiring self-motivation (as opposed to external stimulation) [1]. It has become a major complaint with the fast pace of modern life. Together with stress, it is an important predisposing and perpetuating factor in chronic fatigue [2]. Numerous studies have investigated skeletal muscle metabolism in CF, but few have investigated the changes in cerebral energy production and oxidative function that play vital roles in CF. Although the exact mechanism of CF is unclear, a feeling of low energy is always involved, which suggests that CF is closely related to energy metabolism and oxidative function in the central nervous system [3]. Since the brain relies on aerobic metabolism, cerebral oxygen tension is the basic guarantee of brain function and this process mainly relies on brain mitochondrial function [4].

CF is currently treated with pharmacological and nutritional interventions. The pharmacological interventions mainly involve serotonin reuptake inhibitors and excitatory transmitters [5], but accumulating evidence for a negative correlation between CF and serotonin levels has fostered interest in serotonin receptor antagonists as therapeutic candidates [6, 7]. Amphetamine is a close analogue of dopamine and noradrenaline that could also attenuate CF by enhancing motivation through increased dopaminergic and noradrenergic activity [8]. Nutritional supplements may also treat CF, but the clinical evidence for them is incomplete and relatively unpersuasive. For example, branched-chain amino acids may alleviate CF by competing with tryptophan for brain entry across the blood brain barrier, with a consequent reduction in serotonin receptor activation [9, 10]. Some clinical studies support this hypothesis [11], but others have obtained nonsignificant [12] or negative results [13, 14]. Tryptophan supplementation aggravated CF in animal [15, 16] and human trials [17]. A systematic review found that while some studies suggested that carbohydrates could attenuate CF, there is limited and mixed evidence for a direct effect [18]. Overall, we still lack an effective treatment for CF.

Mitochondria are basic organelles that produce adenosine triphosphate (ATP) through the oxidative phosphorylation (OxPhos) pathway and are much distributed in the brain. Their working efficiency directly affects energy metabolism and oxidative function in the brain, and this process plays an important role in CF. Mitochondrial biogenesis can be defined as the growth and division of pre-existing mitochondria, which is accompanied by variations in number, size, and mass [19]. This process could also be influenced by stress and fatigue. Sirtuin 1 (SIRT1) belongs to the family of nicotinamide adenine dinucleotide-dependent* Sir2*-encoded histone deacetylases, which can activate peroxisome proliferator-activated receptor gamma coactivator-1*α* (PGC-1*α*). PGC-1*α* belongs to the PGC family of transcription coactivators. SIRT1 and PGC-1*α* are the “master regulators” of mitochondrial biogenesis because they co-activate the transcription factors and nuclear receptors that regulate mitochondrial protein expression [20]. Nuclear transcription factor 1 (NRF1) is a downstream SIRT1/PGC-1*α* effector and activates the expression of OxPhos components, mitochondrial transporters, and ribosomal proteins [21]. Mitochondrial biogenesis can be promoted through the activation of SIRT1, which activates PGC1*α*, which in turn activates NRF1. SIRT1, PGC-1*α*, and NRF1 then collectively activate and regulate the mitochondrial biogenesis process [22], which makes these proteins the key stimulators, regulators, and biomarkers of mitochondrial biogenesis. Furthermore, the hippocampus is the first brain region to sustain damage in cognitive and neurological diseases, and mitochondrial function alterations are more marked in the hippocampus than in other brain regions [23]. It is thus necessary to investigate the biomarkers of hippocampal mitochondrial biogenesis during CF.

Traditional Chinese medicine (TCM) holds that CF can be attenuated with* Si-Ni-San* (SNS), an ancient Chinese decoction recorded in the Treatise on Cold Pathogenic Diseases (*Shang Han Lun*) by Zhang Zhongjing during the early 2nd century CE. It traditionally consists of four herbs, bupleurum, white peony, immature bitter orange, and liquorice. However, to enhance its effects, we prepared a modified SNS (MSNS) decoction supplemented with* Eleutherococcus senticosus*,* Astragalus *spp., and* Gardenia jasminoides*, which have been studied for potential fatigue attenuation effects [24, 25]. TCM holds that this decoction can harmonise the liver and spleen, enhance the body's* qi*, and ameliorate CF.

We aimed to evaluate the effects of MSNS by inducing a rat model of CF through sleep deprivation [26] and using it to test the effects of low-dose MSNS (LDM), medium-dose MSNS (MDM), and high-dose MSNS (HDM). The MDM group's dose was twice that of the LDM group, and the HDM group's dose was twice that of the MDM group. We compared the MSNS groups to the CF group consisting of rats that underwent CF induction but only received oral saline administration. For a positive control, we created a coenzyme Q10 (CQ) group that underwent CF induction and received CQ [27], which is an essential component in the ATP-producing mitochondrial electron transport chain and can relieve physical fatigue when orally consumed due to its antioxidant effects [28]. We also created a negative control (CON) group consisting of rats that did not undergo CF induction and received only oral saline administration. After treatment, we used behavioural tests to evaluate the rats for endurance, emotion, and cognitive function. We measured biomarkers for energy metabolism and oxidative stress in the blood and liver, inspected hippocampal mitochondrial ultrastructures, and measured mitochondrial DNA (mtDNA) copy number. We also measured gene and protein expression levels of SIRT1, PGC-1*α*, and NRF1.

## 2. Methods

### 2.1. Drugs

We purchased the ingredients for the MSNS decoction from the Tongrentang Drug Store (Beijing, China). The crude drugs were authenticated by Dr. Xiumei Wu, Professor of Pharmacology. The MSNS contents are listed in Table 2.

The mixed ingredients were decocted with boiling distilled water for 30 min and then filtered. The solution was then freeze-dried under a vacuum, ground into powder (yield: 17.5%), and stored at 4°C. CQ was purchased from Eisai (Tokyo, Japan).

### 2.2. High-Performance Liquid Chromatography (HPLC) and Ultra-Performance Liquid Chromatography Coupled to Electrospray Ionization Tandem Mass Spectrometry (UPLC-ESI-MS) Analysis of MSNS

The HPLC analysis method was modified based on previous study [29]. We analysed the MSNS preparation using an Agilent 1260 HPLC system (Agilent, Santa Clara, CA, USA) with an automatic sample injector, an oven, a diode array detector, and a Gemini chromatographic column (250 × 4.6 mm, 5 *μ*m, 110 Å; Phenomenex, Torrance, CA). We dissolved MSNS powder samples weighing approximately 1 g in either water, 50% methanol, 30% ethanol, 50% ethanol, or 70% ethanol, each at 25 ml volumes. The resulting solutions were weighed, ultrasonicated for 1 h, cooled, and weighed again to determine the final volume as 25 ml with supplementing with solvent if necessary. They were then centrifuged at 3,000* g* for 1 min. The supernatant was collected and filtered with a 0.45 *μ*m filter membrane. The selected separation conditions of 30% ethanol as solvent based on extraction rates and correspondent HPLC spectral resolutions are listed in Table 3, and the overlap HPLC chromatograph of different sample preparation conditions is shown in Figure 9(a).

The UPLC-ESI-MS analysis was performed using an UltiMate3000 super high-performance liquid chromatograph (Dionex, Thermo Fisher Scientific, Waltham, MA USA), a Gemini chromatographic column (Agilent poroshell 120 EC-C18, 150 mm × 3.0 mm, 2.7 *μ*m). The selected separation conditions are listed in Table 4, the mass spectrometric parameters are listed in Table 5, and the positive and negative total ion chromatographs of UPLC-ESI-MS are shown in Figure 10. Furthermore, commercial available reference substances were adopted to elucidate the major components of MSNS, referring to Chinese pharmacopeia. The HPLC chromatograms of MSNS with reference substances as external standards were applied to cross-verify the structure determination based on UPLC-ESI-MS analysis and are shown in Figure 9(b).

### 2.3. Animals

Adult male (weight: 120–130 g) Wistar rats were purchased from Beijing Vital River Laboratory Animal Technology (Beijing, China). The experiments were approved by the Beijing University of Chinese Medicine's Institutional Animal Ethics Committee. All animal procedures were performed in accordance with Chinese legislation on the use and care of laboratory animals. All efforts were made to minimize both animal suffering and the number of animals used.

The animals were placed in a room with a 23 ± 1°C temperature, a 30-40% relative humidity level, 12 h of lighting from 06:00 to 18:00, and ad libitum food and purified water. One week of acclimation was permitted before the experiment.

We randomly divided 60 rats into six groups of ten. These groups included the CON group, which received daily oral gavage doses of saline solution (10 ml/kg); the CF group, which received the same treatment as the CON group; the LDM group, which received 0.5 g/kg of MSNS each day; the MSNS group, which received 1 g/kg of MSNS each day; the HDM group, which received 2 g/kg of MSNS each day; and the CQ group, which received 3 g/kg of CQ each day. The dose of intragastric administration depended on the bodyweight, 1ml for 100g bodyweight. All groups received their assigned administration for 21 days. The MSNS doses were set to approximate human doses.

### 2.4. CF Model Induction

We applied the modified multiple platform method to induce CF through sleep deprivation in all rats except those of the CON group. We placed 15 platforms inside each plastic tank (110 × 60 × 40 cm) and filled the tank with water at 22–25°C to a depth of 1 cm below the platform surfaces. We left each rat in its own tank with a supply of food and drinking water from 18:00 to 08:00 the next morning. We repeated this procedure over 21 consecutive days.

### 2.5. Bodyweight

We recorded each rat's bodyweight at 09:00 daily for 21 days.

### 2.6. Behavioural Tests

We performed all behavioural tests the day after the training ended. Each rat was brought to the testing room at 07:00 and allowed to acclimate to the room for 1 h. We then performed the behavioural tests in the following sequence, OFT, EPM, and WST with 2-h breaks between tests, in order to minimize the effect among the tests. All behavioural measurements were performed with EthoVision XT software (Noldus, Wageningen, the Netherlands). Each rat was only tested once. The test arenas were thoroughly cleaned with 75% ethanol between rats.

### 2.7. WST

The WST was performed with a previously published method [30]. In brief, the rats were forced to swim individually in a plastic pool filled with water at a temperature of 20–22°C and a depth of 60 cm. A tin wire weighing 10% of the rat's bodyweight was attached to the rat's tail base. We recorded the swimming time until the rat became exhausted, as indicated by a failure to rise to the surface to breathe within 10 s. The rats were then removed from the water, dried with a towel, and returned to their cages. The water was replaced between rats.

### 2.8. OFT

The OFT measures animal locomotion, exploration, and anxiety [31]. The open field arena (100 × 100 × 40 cm) was constructed of acrylic with grey walls and a black floor. It was divided into 25 equally sized squares. Each rat was placed in the arena for 5 min, during which we measured the time in the central square, number of square crossings, total distance travelled, longest continuous distance travelled, mean velocity, vertical activity, and grooming behaviours.

### 2.9. EPM

The EPM was constructed as previously described [32] with two open arms and two closed arms (all 30 × 5 × 15 cm) that extended from a central open square (5 × 5 cm). The maze was elevated to a height of 45 cm above the floor. We placed each rat in the EPM for 3 min, during which we measured the ratio of time spent in the open arms to total time spent in the arms, the ratio of open arm entries to the total entries, and the time spent in the central area.

### 2.10. Blood Serum Analysis

The rats were anaesthetised with an intraperitoneal injection of 10% pentobarbital sodium (4 ml/kg) and subsequently sacrificed by rapid decapitation. Blood was collected from the torso in a blood collection tube and subsequently centrifuged at 3,000* g* for 20 min at 4°C to isolate the serum. We assessed CK and LDH activity levels with assay kits from Bioassay Systems (Hayward, CA) and BUN levels with an assay kit from Genmed Scientifics (Shanghai, China). We performed colorimetric readings of these assays with a Multiskan GO microplate reader (Thermo Fisher, St. Louis, MO).

### 2.11. Liver Tissue Analysis

After collecting blood, we immediately dissected two liver pieces from each rat, homogenised them, centrifuged them at 14,000* g* for 5 min at 4°C, and then collected the supernatant. We measured MDA levels and SOD activity with assay kits from BioVision (Milpitas, CA). We again performed colorimetric readings with the Multiskan GO microplate reader.

### 2.12. Transmission Electron Microscope (TEM)

We used TEM to examine hippocampal mitochondria (*n* = 3 per group). After removing the hippocampus, we took three randomly selected 1-mm^3^ pieces from the CA1 region of each rat and immediately fixed them in 2.5% glutaraldehyde (pH = 7.4) for 4 h at 4°C. The samples were then dehydrated and fixed using previously published methods [33]. We examined and photographed the samples with a JEM-1230 device (JEOL, Tokyo, Japan).

### 2.13. Quantitative Reverse Transcription PCR (RT-PCR)

We removed the hippocampus and immediately placed it in liquid nitrogen and then stored as −80°C until assay. We used RT-PCR to quantify the gene expression of cytochrome b,* SIRT1*,* PPARGC1A*,* NRF1*, and, as a control,* ACTB*. We extracted total RNA with the SV Total RNA Isolation System (Z3100, Promega, Madison, WI). We then reverse-transcribed 1 *μ*g of total RNA to 50 *μ*l of cDNA with the Reverse Transcription System (A3500, Promega). We designed primers with Primer-BLAST (https://www.ncbi.nlm.nih.gov/tools/primer-blast/) according to the GenBank mRNA sequences for cytochrome b,* SIRT1*,* PPARGC1A*,* NRF1*, and* ACTB*. The PCR products were run through a 0.8% agarose electrophoresis gel to confirm the expected sizes, and the gel bands were visualised with a FluorChem M gel image analysis system (Alpha Innotech, San Leandro, CA). All results were normalised to the* ACTB* expression. The primer sequences are listed in Table 7. RT-PCR experiments were performed with the CFX96 Touch system (Bio-Rad, Hercules, CA).

The reaction involved initial activation at 95°C for 30 s, 40 cycles of denaturation at 95°C for 5 s and annealing at 60°C for 30 s, and melt curve determination at 65–95°C for 5 s. All measurements were performed in triplicate. The RT-PCR data were represented as Ct values. The relative mRNA expression levels for various genes were determined with the 2-ΔΔCt method [34].

### 2.14. Western Blotting

We collected hippocampal proteins in a radioimmunoprecipitation assay buffer containing phenylmethylsulfonyl fluoride (100:1). Protein concentrations were determined with a bicinchoninic acid assay kit (Solarbo, Beijing, China). Proteins were treated with a sodium dodecyl sulfate- (SDS-) loading buffer after quantitative assessment, subjected to 12% SDS polyacrylamide gel electrophoresis, and then transferred onto polyvinylidene difluoride membranes (5 *μ*l for each sample, 80 V, 100 min). After blocking with 5% dried skim milk for 1 h, the membranes were incubated with anti-SIRT1 (1:2400), anti-PGC-1*α* (1:2000), anti-NRF1 (1:3000), and anti-*β* actin (1:10,000) antibodies (Proteintech, Rosemont, IL) at room temperature for 1 h. They were then incubated with IRDye secondary antibodies (1:10,000; ZSGB-BIO, Beijing, China) for 1 h at room temperature. After the membranes were washed five times with tris-buffered saline-Tween 20 for 25 min, an ECL Prime western blotting detection reagent (GE Healthcare, Chicago, IL) was used to expose protein bands. Protein bands were visualised with a FluorChem M gel image analysis system (Alpha Innotech).

### 2.15. Statistical Analysis

The data were expressed as means ± standard errors. All data were initially tested for normality and homogeneity of variance and then analysed with one-way analysis of variance or the Kruskal–Wallis test. Turkey's test or Mann–Whitney* U* test were used for group comparisons. All data were analysed in SPSS version 17.0 (IBM, Armonk, NY). We defined statistical significance as* P* < 0.05.

Bar graphs were produced with GraphPad Prism 5 (GraphPad, San Diego, CA), and graphs, TEM pictures, and cropped blots were combined in PowerPoint 2012 (Microsoft, Redmond, Washington).

## 3. Results

### 3.1. Effects on Growth

**Figure 1:**
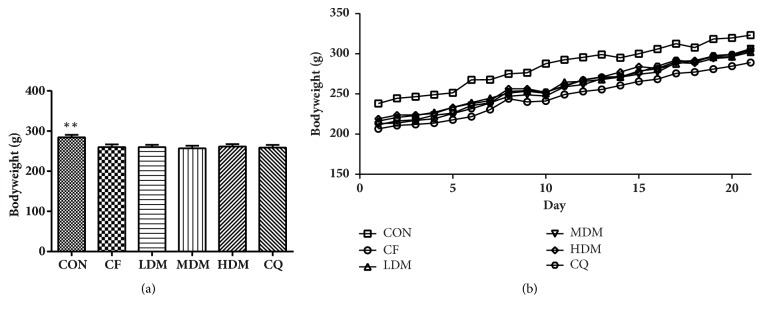
Bodyweight. (a) Bodyweights of different groups. (b) Changes in bodyweights measured daily at 09:00. All data are represented as the mean ± standard error (n = 10 per group). *∗∗*P < 0.01 vs. the CF group. Abbreviations: CON: negative control; CQ: coenzyme Q10; HDM: high-dose modified Si-Ni-San; LDM: low-dose modified Si-Ni-San; MDM: medium-dose modified Si-Ni-San; CF: untreated central fatigue.

We observed significant between-group differences in bodyweight (F_(5, 120)_ = 2.722,* P *< 0.05). The bodyweights of the CF group were significantly lower than those of the CON group (*P* < 0.01) but not significantly different from those of any treatment group (*P* > 0.05). There were no significant between-group differences in bodyweight growth rates (Figure 1).

### 3.2. Weight-Loaded Swimming Test (WST)

**Figure 2:**
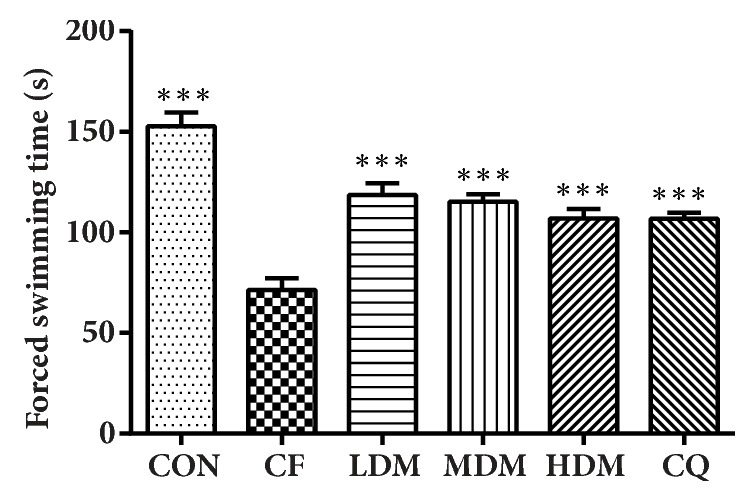
Weight-loaded swimming test endurance. Average swimming endurance is shown for each group. All data are represented as the mean ± standard error (n = 10 per group). *∗∗∗*P = 0.001 vs. the CF group. Abbreviations: CON: negative control; CQ: coenzyme Q10; HDM: high-dose modified Si-Ni-San; LDM: low-dose modified Si-Ni-San; MDM: medium-dose modified Si-Ni-San; CF: untreated central fatigue.

We found significant between-group differences in swimming endurance in the WST (F_(5, 54)_ = 26.364,* P *< 0.001). The CF group's swimming endurance was significantly lower than that of the CON group (*P < *0.001), and all treatments provided some significant recovery (*P *< 0.001), though no significant differences were observed between different treatment groups (*P *> 0.05) (Figure 2).

### 3.3. Open Field Test (OFT)

Table 1 shows the different OFT parameters. We observed significant between-group differences in time spent in the centre (F_(5, 54)_ = 13.497,* P *< 0.001), which is the main indicator of depression-like behaviour, and other depression-indicating parameters including total travel distance (H_(5)_ = 35.993,* P* < 0.001), the number of times the rats crossed between squares (H_(5)_ = 31.226,* P* < 0.001), the longest distance continuously travelled (H_(5)_ = 27.682,* P *< 0.001), mean velocity (H_(5)_ = 33.902,* P < *0.001), total vertical activity time (H_(5)_ = 17.452,* P < *0.01), the number of grooming episodes (H_(5)_ = 15.222,* P < *0.01), and total grooming time (H_(5)_ = 11.14,* P < *0.05). The CF group exhibited several significant differences from the CON group, including more time spent in the centre (*P* < 0.001), greater total travel distances (*P *< 0.001), more square crossings (*P* < 0.001), greater maximum continuous travel distances (*P *< 0.001), greater velocities (*P < *0.001), reduced vertical activity time (*P < *0.001), fewer grooming episodes (*P < *0.05), and reduced grooming time (*P < *0.05), all of which suggest depression. Relative to the CF group, all treatment groups spent significantly less time in the centre (*P *< 0.001), with least significant difference testing revealing that MDM was significantly more effective than the other treatments in reducing centre time (*P *< 0.05). LDM (*P* < 0.01) and MDM (*P *< 0.05) both significantly reversed the CF group's increased total travel distances, with LDM being significantly more effective than MDM (*P *< 0.05). Only LDM significantly reversed increased square crossings (*P *< 0.05). LDM (*P < *0.01) and HDM (*P < *0.05) both significantly reversed the increased maximum continuous travel distances, with LDM being significantly more effective than HDM (*P < *0.05). Only LDM significantly reversed the increased velocities (*P < *0.01). HDM (*P < *0.01) and CQ (*P < *0.01) both significantly restored the lost vertical activity time. LDM (*P < *0.05), MDM (*P < *0.01), and CQ (*P < *0.05) all significantly reversed the decrease in grooming episode numbers. LDM (*P < *0.05) and MDM (*P < *0.01) significantly restored total grooming time.

### 3.4. Elevated Plus Maze (EPM)

**Figure 3:**
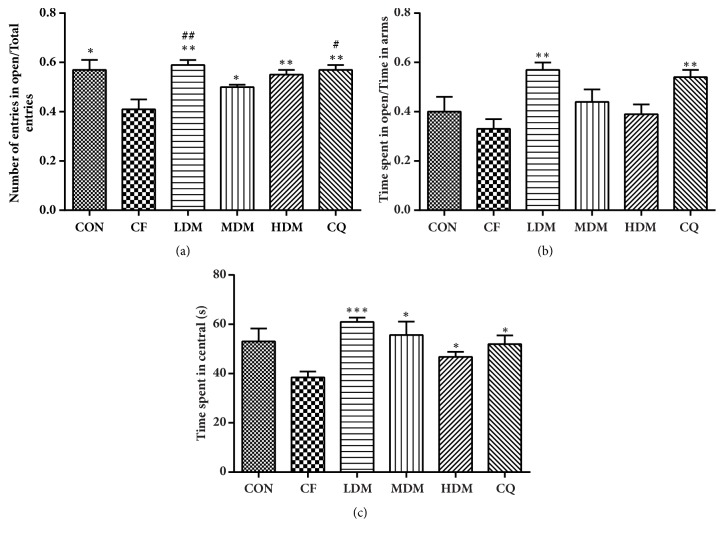
Elevated plus maze assessment. (a) The ratio of open arm entries to total arm entries. (b) Ratio of time spent in the open arm to time spent in all arms. (c) Time spent in the centre of the maze. All data are represented as the mean ± standard error (n = 10 per group). *∗*P < 0.05, *∗∗*P < 0.01, and *∗∗∗*P = 0.001 vs. the CF group; #P < 0.05 and ##P < 0.01 vs. the MDM group. Abbreviations: CON: negative control; CQ: coenzyme Q10; HDM: high-dose modified Si-Ni-San; LDM: low-dose modified Si-Ni-San; MDM: medium-dose modified Si-Ni-San; CF: untreated central fatigue.

The EPM is a classic evaluation of anxiety in rodents. We observed significant between-group differences in the ratio of open arm entries to total arm entries (H_(5)_ = 19.411,* P < *0.01; Figure 3(a)), with the CF group exhibiting significantly lower ratios than the CON group did (*P < *0.05). All treatments significantly reversed this effect, with LDM (*P < *0.01) and CQ (*P < *0.05) both being significantly more effective than MDM. We also observed significant between-group differences in the ratio of time spent in the open arm to the total time spent in all arms (H_(5)_ = 20.82,* P < *0.01; Figure 3(b)), but the CF group exhibited ratios that were nonsignificantly different (*P > *0.05) from those of the CON group. However, LDM (*P < *0.01) and CQ (*P < *0.01) did significantly increase the time spent in the open arms. Time spent in the centre area is an indicator of capacity for analysis and judgment, and we again observed significant between-group differences (H_(5)_ = 18.324,* P < *0.01; Figure 3(c)), but the CF group's time in the centre was only nonsignificantly less than that of the CON group (*P > *0.05). However, all treatments significantly increased time spent in the centre.

### 3.5. Blood Serum Biomarkers

**Figure 4:**
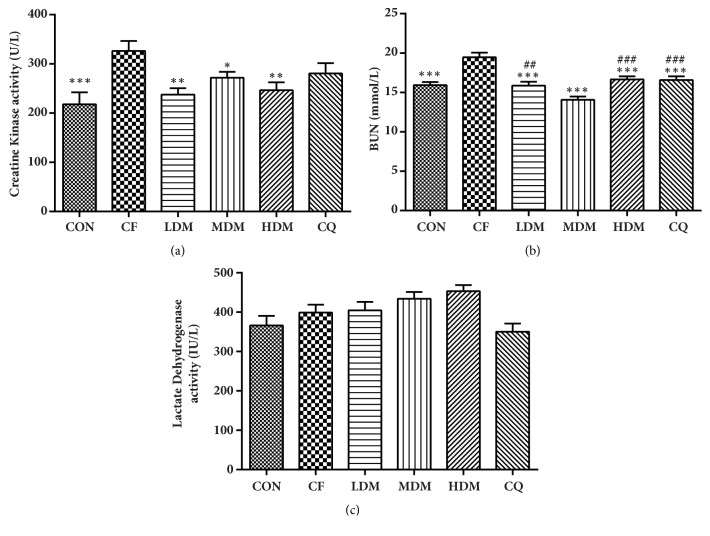
Analysis of blood serum biomarkers. All data are represented as the mean ± standard error (n = 10 per group). *∗*P < 0.05, *∗∗*P < 0.01, and *∗∗∗*P = 0.001 vs. the CF group; ##P < 0.05 and ###P = 0.001 vs. the MDM group. Abbreviations: CON: negative control; CQ: coenzyme Q10; HDM: high-dose modified Si-Ni-San; LDM: low-dose modified Si-Ni-San; MDM: medium-dose modified Si-Ni-San; CF: untreated central fatigue.

We detected significant between-group differences in creatine kinase (CK) activity (F_(5, 54)_ = 4.362,* P < *0.01; Figure 4(a)), blood urea nitrogen (BUN) levels (F_(5, 54)_ = 14.056,* P < *0.001; Figure 4(b)), and lactate dehydrogenase (LDH) activity (H_(5)_ = 14.205,* P < *0.05; Figure 4(c)). Compared to the CON group, the CF group exhibited significantly elevated CK activity (*P < *0.001) and BUN levels (*P < *0.001). LDM, MDM, and HDM all significantly reversed the CF group's elevated CK activity, but CQ achieved only a nonsignificant reduction (*P > *0.05). All treatments significantly reversed the CF group's elevated BUN levels (*P < *0.001), with MDM being significantly more effective than any other treatment (*P < *0.001). Least significant difference testing indicated that no two groups exhibited significantly different LDH activity levels, but the MSNS groups seemed to exhibit elevated LDH activity relative to the CON group, whereas the CQ group seemed to exhibit decreased activity.

### 3.6. Malondialdehyde (MDA) and Superoxide Dismutase (SOD) in Liver Tissues

**Figure 5:**
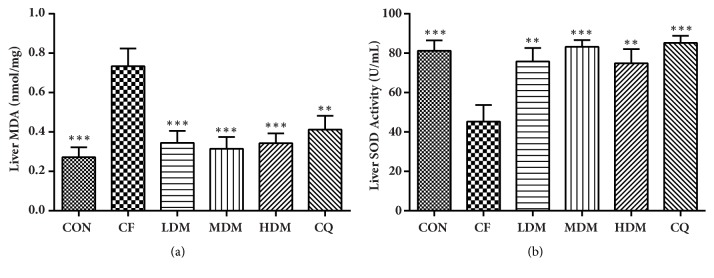
Analysis of the liver MDA levels and SOD activities. All data are represented as the mean ± standard error (n = 7 per group). *∗∗*P < 0.01 and *∗∗∗*P = 0.001 vs. the CF group. Abbreviations: CON: negative control; CQ: coenzyme Q10; HDM: high-dose modified Si-Ni-San; LDM: low-dose modified Si-Ni-San; MDM: medium-dose modified Si-Ni-San; CF: untreated central fatigue.

We detected significant between-group differences in hepatic MDA levels (F_(5, 36)_ = 0.173,* P < *0.001) and hepatic SOD activity (F_(5, 36)_ = 5.907,* P < *0.001) (Figure 5). Compared to the CON group, the CF group exhibited significantly greater MDA levels (*P < *0.001) and significantly lower SOD activity (*P < *0.001), and all treatments significantly reversed these effects.

### 3.7. Ultrastructural Changes in the Hippocampal CA1 Region

**Figure 6:**
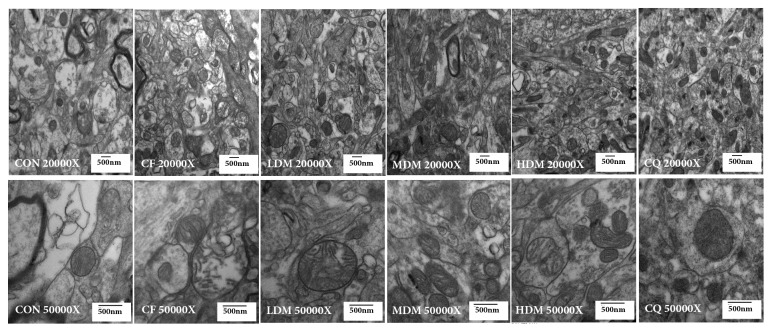
Representative transmission electron microscope photos of hippocampal CA1 region mitochondria. Each group was observed at 20,000× and 50,000× magnifications to detect changes in the hippocampal mitochondria. Abbreviations: CON: negative control; CQ: coenzyme Q10; HDM: high-dose modified Si-Ni-San; LDM: low-dose modified Si-Ni-San; MDM: medium-dose modified Si-Ni-San; CF: untreated central fatigue.

Representative ultrastructural micrographs of hippocampal CA1 region mitochondria for each group are shown in Figure 6. The mitochondrial membrane was smooth and clear in the CON group, with mitochondrial cristae that were clearly visible and properly ordered. Compared to the CON group, the CF group exhibited fewer mitochondria and several degenerative changes including blurred external membranes of the intracellular mitochondria, swollen cristae, numerous irregular mitochondria, and the absence of part of the mitochondrial membrane. All treatments reversed these alterations.

### 3.8. mtDNA Copy Number and mRNA Gene Expression of SIRT1, PGC-1*α*, and NRF1

**Figure 7:**
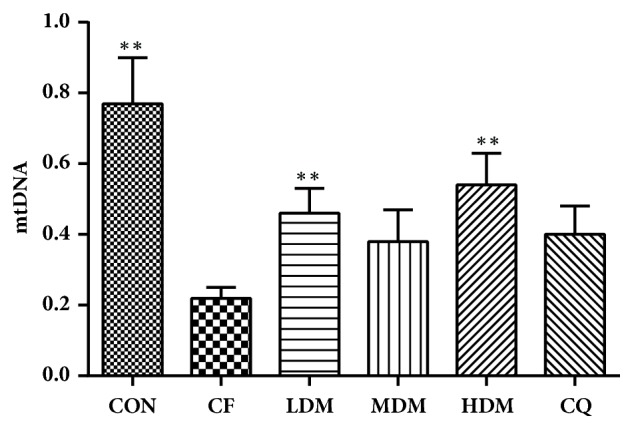
mtDNA. All data are represented as the mean ± standard error (n = 7 per group). *∗∗*P < 0.01 vs. the CF group. Abbreviations: CON: negative control; CQ: coenzyme Q10; HDM: high-dose modified Si-Ni-San; LDM: low-dose modified Si-Ni-San; MDM: medium-dose modified Si-Ni-San; mtDAN: mitochondrial DNA; CF: untreated central fatigue.

The mtDNA copy number was calculated from the cytochrome b △CT value relative to that of beta actin. We observed significant between-group differences in mtDNA copy number (H_(5)_ = 14.777,* P < *0.05). The CF group exhibited significant reductions in mtDNA copy number relative to the CON group (*P < *0.01). This reduction was significantly reversed by LDM (*P < *0.05) and HDM (*P < *0.01) and nonsignificantly reversed by MDM and CQ (Figure 7).

**Figure 8:**
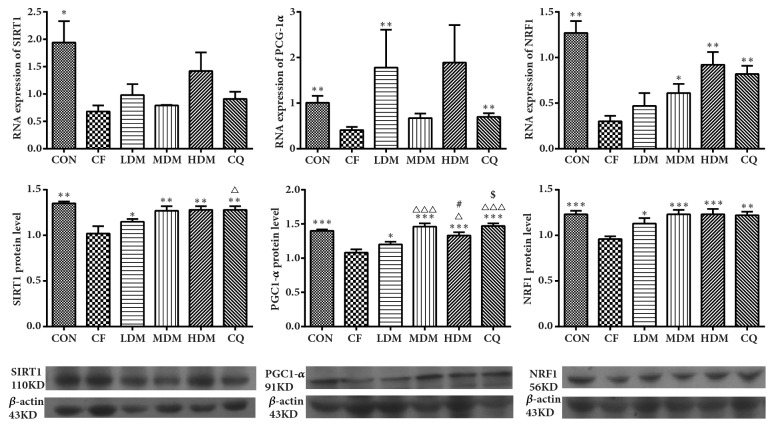
Expression of SIRT1, PGC-1*α*, and NRF1. Representative cropped blots are exhibited, and the full-length blots are presented in Supplementary Figure (available here). All data are represented as the mean ± standard error (n = 7 per group). *∗*P < 0.05, *∗∗*P < 0.01, and *∗∗∗*P = 0.001 vs. the CF group. #P < 0.05 and ###P = 0.001 vs. the MDM group. △P < 0.05 and △△△P < 0.001 vs. the LDM group. $P < 0.05 vs. the HDM group. Abbreviations: CON: negative control; CQ: coenzyme Q10; HDM: high-dose modified Si-Ni-San; LDM: low-dose modified Si-Ni-San; MDM: medium-dose modified Si-Ni-San; NRF1: nuclear respiratory factor 1; PGC-1*α*: peroxisome proliferator-activated receptor gamma coactivator-1*α*; SIRT1: sirtuin 1; CF: untreated central fatigue.

Real-time polymerase chain reaction (PCR) revealed significant between-group differences in the hippocampal mRNA expression of* SIRT1 *(H_(5)_ = 11.305,* P < *0.05), the PGC-1*α*–encoding* PPARGC1A* (H_(5)_ = 12.305,* P < *0.05), and* NRF1* (H_(5)_ = 25.524,* P < *0.001). Relative to the CON group, the CF group exhibited significantly decreased mRNA expression of* SIRT1* (*P < *0.05),* PPARGC1A* (*P < *0.01), and* NRF1* (*P < *0.01). No treatment provided significant recovery of* SIRT1 *expression (*P > *0.05). Only LDM (*P < *0.01) and CQ (*P < *0.01) provided significant recovery of* PPARGC1A* expression. All treatments except for LDM provided significant recovery of* NRF1* expression (*P < *0.05) (Figure 8).

Western blotting revealed significant between-group differences in the protein expression levels of SIRT1 (H_(5)_ = 23.578,* P < *0.001), PGC-1*α* (F_(5, 36)_ = 14.052,* P < *0.001), and NRF1 (F_(5, 36)_ = 4.865,* P < *0.01). Compared to the CON group, the CF group exhibited significantly lower levels of SIRT1, PGC-1*α*, and NRF1. All treatments provided significant recovery of SIRT1 expression (*P < *0.01), with CQ being significantly more effective than LDM (*P < *0.05). All treatments provided significant recovery of PGC-1*α* expression, with MDM and CQ being significantly more effective than LDM or HDM. All treatments provided significant recovery of NRF1 expression (Figure 8).

### 3.9. HPLC and UPLC-ESI-MS Analysis

**Figure 9:**
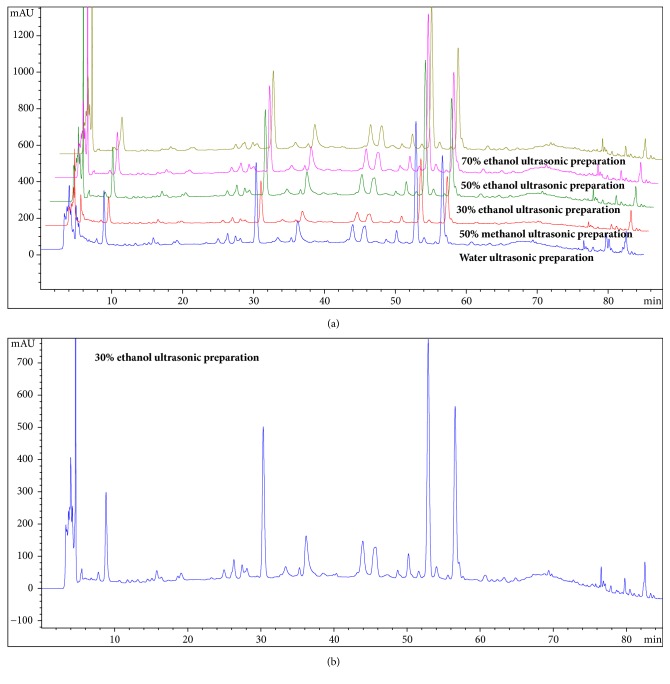
HPLC analysis of MSNS. (a) Overlap chromatograms of the different sample preparation conditions. (b) The chromatograms of MSNS and reference substances as external standards dissolved in 30% ethanol. 1. Chlorogenic acid, 2. geniposide, 3. paeoniflorin, 4. liquiritin, 5. naringin, 6. monoammonium glycyrrhizinate (ammonium glycyrrhizinate), 7. glycyrrhetinic acid. Abbreviations: HPLC: high-performance liquid chromatography; MSNS: modified Si-Ni-San.

**Figure 10:**
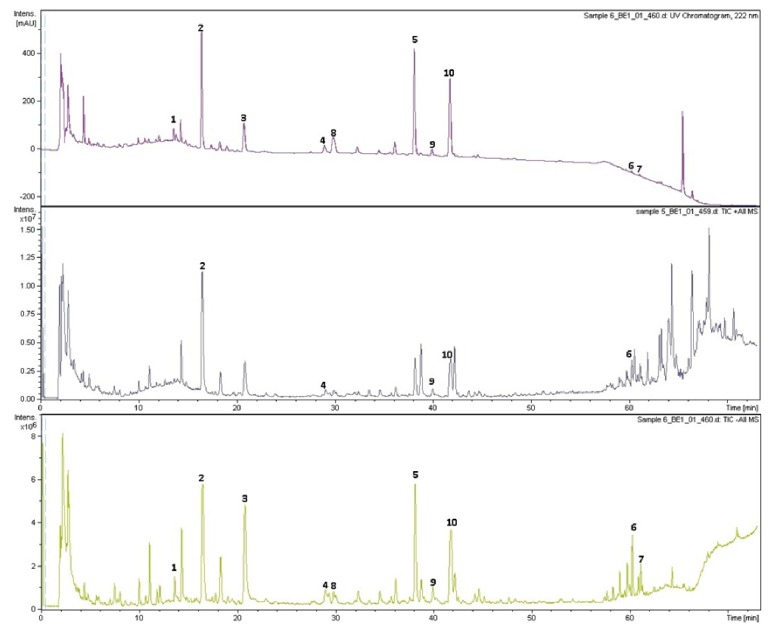
The positive and negative total ion chromatograms of MSNS in UPLC-ESI-MS. Top: UV chromatogram (222 nm), Middle: positive total ion chromatogram, and Down: negative total ion chromatogram. 1. Chlorogenic acid, 2. geniposide, 3. paeoniflorin, 4. liquiritin, 5. naringin, 6. monoammonium glycyrrhizinate (ammonium glycyrrhizinate), 7. glycyrrhetinic acid, 8. liquiritigenin-4'-apiosyi-giucoside (or licuraside/genipin 1-gentiobioside), 9/10. hesperidin (or neohesperidin/rutinum).

We analysed the sample of MSNS using different conditions and found that the 30% ethanol ultrasonic preparation was the best condition to collect the sample of MSNS. The overlap chromatograms of different sample preparation condition chromatograms of HPLC was shown in Figure 9(a). The positive and negative total ion chromatograms of MSNS in UPLC-ESI-MS were shown in Figure 10. The HPLC chromatograms of reference substances and MSNS were shown in Figure 9(b). The main identified components in MSNS were listed in Table 6. Based on these results, the MSNS decoction contains* chlorogenic acid* (peak 1)*, geniposide* (peak 2)*, paeoniflorin* (peak 3)*, liquiritin* (peak 4)*, naringin* (peak 5)*, monoammonium glycyrrhizinate *(*ammonium glycyrrhizinate*) (peak 6)*, glycyrrhetinic acid* (peak 7). Besides,* liquiritigenin-4'-apiosyi-giucoside *(or* licuraside / genipin 1-gentiobioside*) and* hesperidin* (or* neohesperidin / rutinum*) may be the composites of peak 8 and peak 9/10, respectively; meanwhile, further investigation with advanced techniques like MS-MS is needed to testify the existence of these isomers and to elucidate their structures.

## 4. Discussion

CF is a complex state that can be induced by strenuous physical or mental tasks and can result in cognitive dysfunction, decreased self-motivation, decreased physical endurance, negative emotions, and various metabolic disorders in both the central and the peripheral systems. Accumulating evidence suggests that the hippocampal mitochondrial function can directly influence CF by affecting energy metabolism and oxidative processes. We investigated the efficacy of MSNS for alleviating CF induced in rats through long-term intermittent sleep deprivation [35], which can damage physiological functions by affecting metabolic energy [36]. We found that inducing CF promotes negative emotions, physiological dysfunction, and cognitive dysfunction and that MSNS can reverse these effects. CF also caused morphological degeneration of hippocampal mitochondria and decreased expression levels of mitochondrial biogenesis biomarkers, but these effects can also be reversed by administering MSNS. In addition, low-dose and middle-dose MSNS were more effective than high-dose.

One of the most direct and evident consequences of CF is reduced physical activity, which presented as reduced swimming endurance in this study. The WST is frequently used to assess physical endurance in fatigue research, and our WST results indicated that MSNS and CQ enhanced swimming endurance. This is consistent with their antifatigue properties, especially in increasing muscular capacity for force and sustained activity. CF can slow bodyweight gains by reducing appetite and increasing energy consumption, but no treatment increased bodyweight in this study. Further research may be necessary to explain this result.

CF can directly reduce muscular strength and efficiency by slowing oxidative processes and the consumption of energy metabolism products. MSNS can improve these functions, and we observed changes in relevant biomarkers. BUN levels, LDH activity, and CK activity are three important parameters in body function evaluation. BUN levels reflect protein metabolism, whereas LDH and CK activity reflect muscular function and cellular damage [37]. CK and LDH are the main regulatory enzymes of the ATP-phosphocreatine system and sugar metabolism, respectively [38]. Catabolic metabolism of proteins and amino acids increases when sugars and fats provide insufficient energy [39]. All treatments in our study decreased BUN levels, with MDM being notably more effective than the other treatments. All MSNS doses decreased CK activity, but CQ did not, which suggests that MSNS provides better protection and recovery of muscle function during CF than CQ does. However, no treatments were effective in restoring LDH activity, possibly because of sugar metabolism not fully participating in CF in this model. Elevated oxidant levels can weaken muscles and accelerate fatigue [40]. SOD is an important enzyme in antioxidant regulation, and growing evidence suggests that it protects against oxidative stress [41]. It can downregulate lipid peroxidation to maintain the balance of oxidation and antioxidants during strenuous work. MDA levels may reflect oxidative damage because MDA is a product of lipid peroxidation and prostaglandin biosynthesis [42]. Our CF group exhibited increased MDA levels and decreased SOD activity, which suggests oxidative damage during CF. MSNS and CQ both reversed these changes with equal efficacy and no apparent dose-dependency for MSNS. We therefore suggest that MSNS-induced CF alleviation may be related to antioxidant effects.

Negative emotions are another manifestation of CF [43]. The OFT is widely accepted as a reliable test of locomotor activity associated with negative emotions in rodents [44]. The CF group's increased time spent in the centre indicates damaged spatial cognitive function, but all treatments reversed this effect. The mental and physical states of rodents are reflected in such OFT parameters as total distance travelled, number of square crossings, longest continuous travel distance, and mean velocity. Several studies have observed reduction of these parameters in fatigued rats [45, 46]. However, we observed increased locomotor activity in the CF group, probably because while our model does generate physical weakness, it also promotes an anxious and excitable mental state, which is closely related to central rather than peripheral fatigue. Decreases in vertical activity and grooming both reflect anxiety in this test, and we observed evident decreases in the CF group, while all treatments alleviated these effects. Another important negative emotion is anxiety, for which the EPM is a classic test in rodents. The CF group exhibited signs of anxiety such as fewer visits to the open arms, and LDM and CQ reversed these effects. Another important EPM parameter is the time spent in the central area, which can also reflect the decision-making abilities and cognitive function that play a crucial role in CF [47]. Notably, all treatments increased the time spent in the central area. The OFT and EPM results generally suggest negative emotions and cognitive dysfunctions in the CF group that were alleviated by every treatment.

Mitochondrial biogenesis directly affects mitochondrial function, which can be inferred from changes in mitochondrial structures. The morphological changes in the transmission electron microscopy pictures show mitochondrial dysfunction in the hippocampus, with the CF group's hippocampal CA1 regions exhibiting degenerative changes including mitochondrial swelling, broken membranes, and broken and disappearing cristae. These mitochondrial dysfunctions suggest a low efficiency of mitochondrial biogenesis, as does the decreased mtDNA copy number. Nuclear-encoded proteins are essential for mtDNA replication, so their expression is an effective index of mitochondrial biogenesis. All treatments increased mtDNA copy number, which suggests enhancement of mitochondrial biogenesis. Finally, the CF group exhibited decreased gene and protein expression levels of SIRT1, PGC-1*α*, and NRF1, while all treatments restored the expression levels, which suggests that MSNS can alleviate CF-associated hippocampal mitochondrial dysfunction by increasing the efficiency of mitochondrial biogenesis. Furthermore, CQ is an essential component in the mitochondrial electron transport chain and so can enhance mitochondrial function. Notably, MSNS increased expression levels for the proteins of interest with efficacy comparable to that of CQ, which serves as evidence that MSNS can improve mitochondrial function.

Our study therefore suggests that MSNS and CQ can alleviate CF in rats and LDM and MDM were more effective than HDM. The metabolic function and efficiency of MSNS in rats were different from human and vary in different decoction. In the current study, we found that the low dose and middle dose were both more effective than high dose; the reason may refer to the metabolic and absorb efficiency of the decoction in rats. The effects of MSNS manifest in several ways, such as decreasing the levels of energy metabolic products, exerting antioxidant effects, enhancing physical endurance, relieving negative emotions, improving cognitive function, mitigating ultrastructural degenerative changes in mitochondria, and promoting mitochondrial biogenesis in the hippocampus. According to the previous study, alkaloids, flavonoids and triterpenoids comprised majority of compounds in SNS [44]. The MSNS was a modified decoction of SNS, with other 3 herbs added,* Eleutherococcus senticosus, Astragalus spp., and Gardenia jasminoides*, to enhance the effect of antifatigue. Combined with the effect of SNS, the MSNS could be used as a decoction to relieve central fatigue. The components of MSNS include* chlorogenic acid, geniposide, paeoniflorin, liquiritin, naringin, monoammonium glycyrrhizinate *(*ammonium glycyrrhizinate*)*, glycyrrhetinic acid*, etc. The main effective components of MSNS still need further research. Together, these results indicate that the MSNS decoction can alleviate CF by balancing energy metabolism in CNS and providing antioxidant effects. The mechanism appears to be enhancement of hippocampal mitochondrial biogenesis. Our results provide a rationale for human trials of MSNS for treating CF. Further studies are needed to elucidate the underlying mitochondrial molecular mechanism.

## Figures and Tables

**Table 1:** Assessment of open field test. All data were represented as the mean ±SEM (n=10), *∗* refers to P <0.05, *∗∗*refers to P <0.01, and *∗∗∗* refers to P =0.001 vs. CF group. △ refers to P <0.05 vs. LDM group. # refers to P <0.05 vs. MDM group. Abbreviations: CON: negative control; CQ: coenzyme Q10; HDM: high-dose modified Si-Ni-San; LDM: low-dose Si-Ni-San; MDM: medium-dose Si-Ni-San; CF: untreated central fatigue.

**Parameters/group**	**CON**	**CF**	**LDM**	**MDM**	**HDM**	**CQ**
Time spent in central area(s)	16.34 ± 2.38*∗∗∗*	56.98 ± 6.38	32.88 ± 3.3*∗∗∗*^#^	20.22 ± 3.39*∗∗∗*	34.5 ± 3.06*∗∗∗*^#^	31.7 ± 3.49*∗∗∗*^#^

Total distance travelled(cm)	1723.37 ± 74.97*∗∗∗*	3207.36 ± 137.62	2400.78 ± 140.16*∗∗*^#^	2781.9 ± 80.26*∗*	2938.82 ± 114.8	3290.7 ± 183.31

Number of crossing squares	65 ± 5.51*∗∗∗*	147.2 ± 7.97	113.2 ± 8.42*∗*	114.7 ± 9.47	125.8 ± 7.08	147.9 ± 6.04

Maximum continuous distance(cm)	9.13 ± 0.37*∗∗∗*	12.9 ± 0.5	10.64 ± 0.34*∗∗*	11.91 ± 0.67	11.54 ± 0.16*∗*^△^	11.6 ± 0.35

Mean velocity(cm/s)	6.95 ± 0.22*∗∗∗*	10.7 ± 0.46	8.35 ± 0.36*∗∗*	9.67 ± 0.26	9.81 ± 0.39	10.48 ± 0.42

Vertical activity(s)	33.14 ± 2.08*∗∗∗*	19.09 ± 1.97	25.88 ± 2.61	25.92 ± 3.28	33.98 ± 2.97*∗∗*	29.94 ± 2.31*∗∗*

Number of grooming behavior	2.3 ± 0.54*∗*	1 ± 0.26	2.5 ± 0.4*∗*	2.6 ± 0.31*∗∗*	1.4 ± 0.34	2.5 ± 0.43*∗*

Time of grooming behavior(s)	7.4 ± 2.36*∗*	2.69 ± 1.05	6.91 ± 1.8*∗*	8.15 ± 1.22*∗∗*	4.71 ± 1.23	4.2 ± 1.31

**Table 2:** Contents of Modified Si-Ni-San. The components of MSNS, the general profile of each herb, and the ratio were presented in the table.

**Chinese name**	**Botanical name**	**Common name**	**Family**	**Weight(g)**	**Part used**
Chai Hu	Bupleurum Chinense DC	BUPLEURI RADIX	Umbelliferae	12	root

Bai Shao	Paeonia lactiflora Pall	PAEONIAE RADIX ALBA	Ranunculaceous	10	root

Zhi Qiao	Citrus aurantium L	AURANTII FRUCTUS	Rutaceae	8	Fruit

Gan Cao	Glycyrrhiza uralensis Fisch	GLYCYRRHIZAE RADIX ET RHIZOMA	Leguminosae	8	root and rhizome

Ci Wu Jia	Acanthopana.z senticosus ( Rupr.et Maxim.) Harms	ACANTHOPANACIS SENTICOSI RADIX ET RHIZOMA SEU CAULIS	Araliaceae	10	root and rhizome

Huang Qi	Astragalus membranaceus (Fisch.) Bge. var. mongholicus ( Bge. ) Hsiao or Astragalus membranaceus (Fisch.) Bge	ASTRAGALI RADIX	Leguminosae	8	root

Zhi Zi	Gardenia jasminoides Ellis	GARDENIAE FRUCTUS	Rubiaceae	8	fruit

**Table 3:** Selected separation condition of HPLC. The sample of MSNS was analysed by HPLC; the selected separation condition was presented in the table.

Mobile phase	Gradient condition	Flow rate	Capillary temperature	Injection volume	Detection wavelength
A: phosphate buffer (PH=2)	0~5min: 5%~5%B;	0.8ml/min	35°C	10*μ*L	222nm
	5~30min:5%~15%B;				
B: acetonitrile	30~60min:15%~25%B;				
	60~70min:25%~40%B;				
	70~80min:40%~95%B;				
	80~85min:95%~95%B				

**Table 4:** Selected separation condition of UPLC-ESI-MS. The sample of MSNS was analysed by UPLC-ESI-MS; the selected separation condition was presented in the table.

Mobile phase	Gradient condition	Flow rate	Capillary temperature	Injection volume	Detection wavelength
A: 0.05%formic acid-water	0~4.5min: 5%~5%B;	0.3ml/min	35°C	2.6*μ*L	222nm
	4.5~10.42min:5%%~13%B;				
	10.42~21.42min:13%%~13%B;				
	21.42~53.42 min,13%%~29%B;				
B: acetonitrile	53.42~59.87 min,29%%~66%B;				
	59.87~63.87 min,66%%~95%B;				
	63.87~73 min,95%%~95%B				

**Table 5:** Mass spectrometric parameters of UPLC-ESI-MS.

Source Type	Scan Range	Ion Polarity	Nebulizer Gas Pressure	Capillary Voltage	Dry Gas Flow Rate
ESI	50-1500m/z	Positive	2.0Bar	4500V	200°C
		Negative		4500V	

**Table 6:** The identification of main compounds in MSNS.

**peak**	**retention time/min**	**relative molecular mass**	**chemicals**	cation			anion			
				**[M+H]** ^**+**^	**[2M+H]** ^**+**^	**[M-NH**_**3 **_ **+H]** ^**+**^	**[2M-H]** ^−^	**[M-H]** ^−^	**[M+HCOOH-H]** ^−^	**[M-NH**_**3**_ **-H]** ^−^
1	13.6	354.31	Chlorogenic acid				707.2299	353.1308		

2	16.4	388.37	Geniposide		777.6093		775.3155	387.1745	433.1810	

3	20.8	480.46	Paeoniflorin					479.2033	525.2093	

4	29	418.3	Liquiritin		837.7795			417.1674		

5	38.2	580.54	naringin				1159.4040	579.2242		

6	60.3	839.96	monoammonium glycyrrhizinate (ammonium glycyrrhizinate)			823.8823				821.4439

7	61.1	470.68	glycyrrhetinic acid					469.2328	515.2390	

8	29.8	550	liquiritigenin-4'-apiosyi-giucoside / licuraside/ genipin 1-gentiobioside				1099.3811	549.2121	595.2191	

9	40.0	610	hesperidin / neohesperidin / rutinum	611.0467	1223.2159		1219.4282	609.2350		

10	41.8	610	hesperidin / neohesperidin / rutinum	611.0461	1223.2104		1219.4251	609.2348		

**Table 7:** .The primers for real-time RT-PCR. The primers of *β*-actin (as control), cytochrome b, PGC-1*α*, SIRT1, and NRF1 were presented in the table.

**genes**	**forward**	**reverse**
*β*-actin	5′-CTTAGAAGCATTTGCGGTGCCGATG-3′	5′-TCATGAAGTGTGACGTTGCATCCGT-3′
Cytochrome b	5′-AATTCTCCGATCCGTCCCTA-3′	5′-GGAGGATGGGGATTATTGCT-3′
PGC-1*α*	5′-GCGGACAGAACTGAGAGACC-3′	5′-CGACCTGCGTAAAGTATATCCA-3′
SIRT1	5′-CCTGACTTCAGATCAAGAGATGGTA-3′	5′-CTGATTAAAAATATCTCCACGAACAG-3′
NRF1	5′-TTGGAGAATGTGGTGCGTAAGT-3′	5′-GAGAGGCGGCAGTTCTGAGT-3′

## Data Availability

The data used to support the findings of this study are available from the corresponding author upon request.

## Supplementary Materials

Supplementary Materials The full-length picture of western blot.
