# Supplementary figures and images for: Modified Si-Ni-San Decoction Ameliorates Central Fatigue by Improving Mitochondrial Biogenesis in the Rat Hippocampus
Source: Evid Based Complement Alternat Med. 2018 Jul 29;2018:9452127. doi: 10.1155/2018/9452127 (PMC6087596; doi:10.1155/2018/9452127)

**Supplementary figure.** Full length picture of western blot.


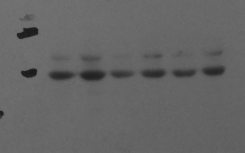
β-actin


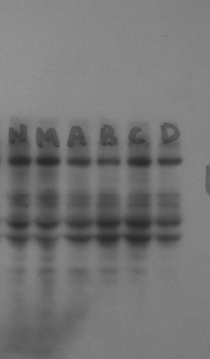


SIRT1:


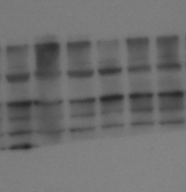


PGC1-α：


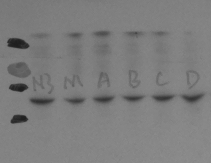


NRF1:

Supplement: Supplementary Materials — The full-length picture of western blot. [file 9452127.f1.docx]
